# Supplementary material for: Smart Dura: a functional artificial dura for multimodal neural recording and modulation
Source: bioRxiv. 2025 Jul 24:2025.02.26.640369. Preprint. [Version 2] doi: 10.1101/2025.02.26.640369 (PMC12330592; doi:10.1101/2025.02.26.640369)
Supplement: Supplement 1 [file NIHPP2025.02.26.640369v2-supplement-1.pdf]

bioRxiv preprint doi: https://doi.org/10.1101/2025.02.26.640369; this version posted July 24, 2025. The copyright holder for this preprint (which was not certified by peer review) is the author/funder, who has granted bioRxiv a license to display the preprint in perpetuity. It is made available under aCC-BY 4.0 International license.

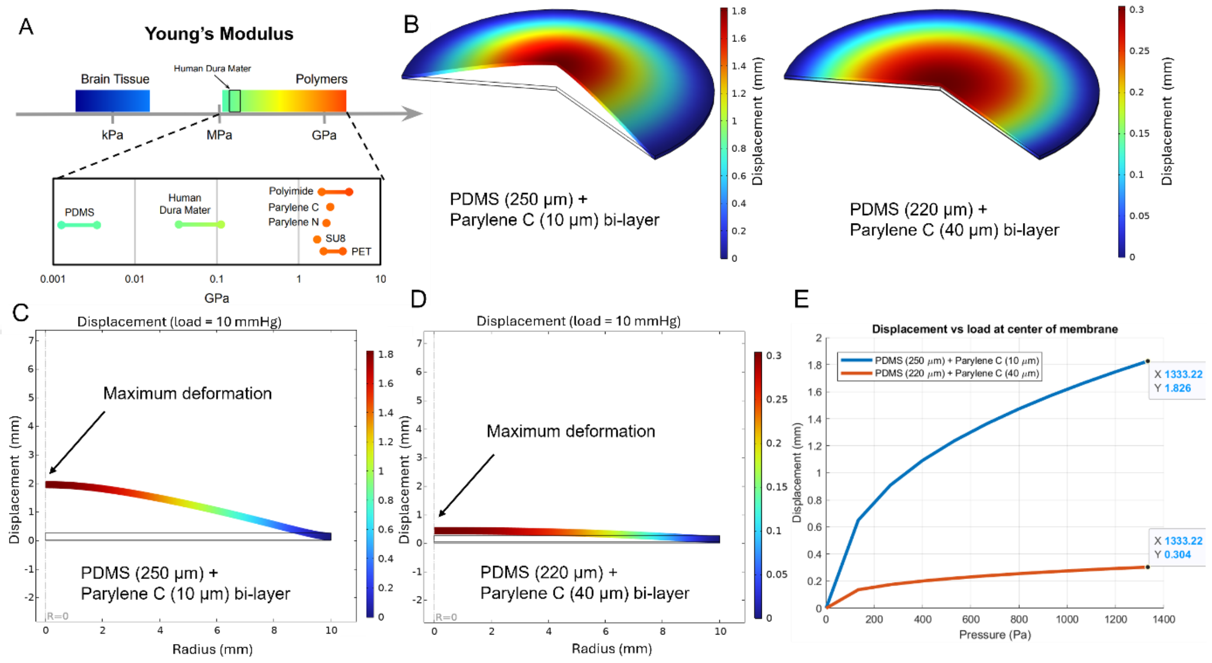


**Figure S1. Material choice and tunable mechanical properties of Smart Dura.** A) Young's moduli of polymers used for neural interfaces, brain tissue, and native dura. Figure modified with permission from [25]. B) Comparison of the 3D deformation profile of 250 µm and 10 µm (left) and 220 µm and 40 µm (right) PDMS/Parylene C bi-layer substrates with 10 mmHg uniform pressure load applied to the bottom. C) Cross-section 2D plot of the deformation from the center of the membrane to its edge for the 250 µm and 10 µm PDMS-Parylene C bi-layer. D) Cross-section 2D plot of the deformation from the center of the membrane to its edge for the 220 µm and 40 µm PDMS-Parylene C bi-layer. Note the notable difference in deformation compared to C). E) Comparison of displacement at the center of the membrane of the two difference thickness ratio PDMS-Parylene C bi-layers subject to different nominal cranial pressure loads (1-10 mmHg or 133.32-1333.2 Pa), showing the capability of tuning the mechanical stiffness of the bulk bi-layer substrate based on the relative thickness ratio of PDMS and Parylene C.

bioRxiv preprint doi: https://doi.org/10.1101/2025.02.26.640369; this version posted July 24, 2025. The copyright holder for this preprint (which was not certified by peer review) is the author/funder, who has granted bioRxiv a license to display the preprint in perpetuity. It is made available under aCC-BY 4.0 International license.

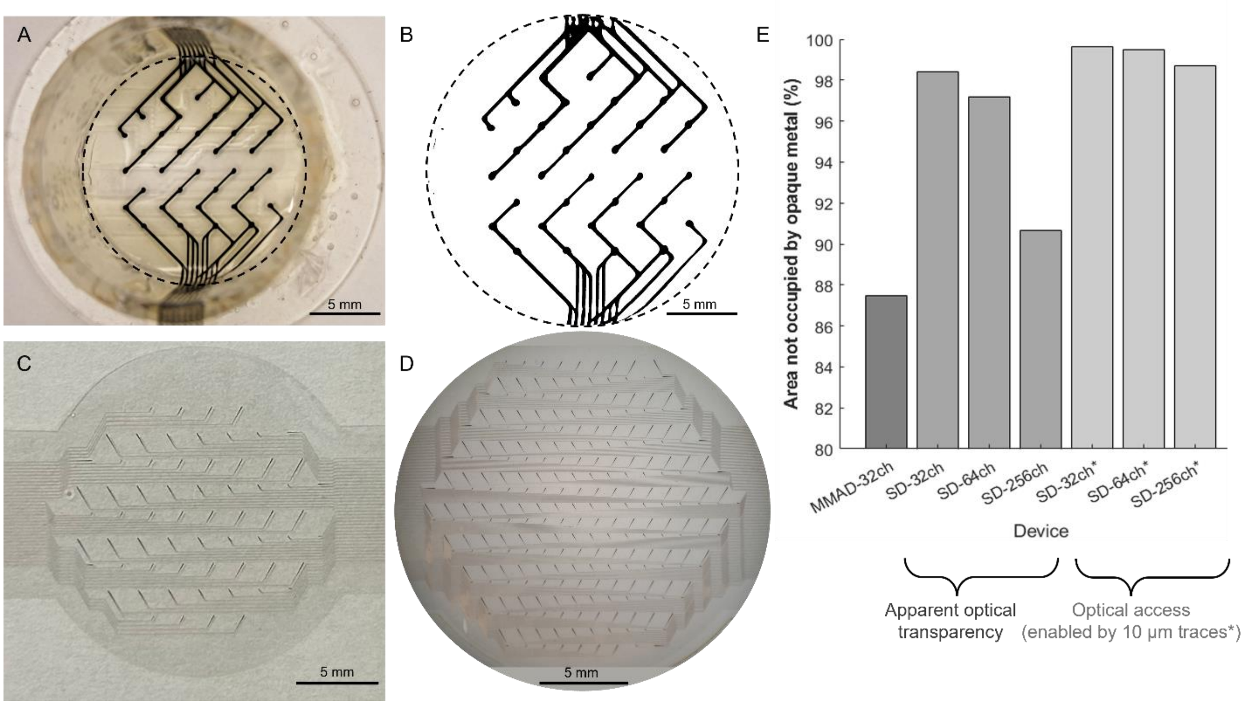


**Figure S2. Comparison of optical window areas.** A) Photograph of 32-channel MMAD with 0.5 mm diameter electrodes and 250-300 µm wide traces. B) Binary image of the optical window of the MMAD used to calculate the percentage of white (transparent) vs black (opaque) pixels to obtain the percentage of area of the MMAD optical window not blocked by the opaque metals and electrodes. C) Photograph of 64-channel Smart Dura over a white background. D) Photograph of the high-density 256-channel Smart Dura over a white background. E) Bar plot comparing the percentage of the optical window area not blocked by opaque electrodes and traces, where SD stands for Smart Dura. The 32-channel MMAD has an optical access of 87.46% while the Smart Dura has optical access for the 32-channel, 64-channel, and 256-channel of 98.42%, 97.19%, and 90.68% respectively, demonstrating the improved optical access of the Smart Dura provided by the microfabrication techniques described in this paper. If 10 µm trace width features are further neglected in Smart Dura designs as discussed above, the optical access for the 32-channel, 64-channel, and 256-channel increases to 99.64%, 99.46%, and 98.69% respectively.

bioRxiv preprint doi: https://doi.org/10.1101/2025.02.26.640369; this version posted July 24, 2025. The copyright holder for this preprint (which was not certified by peer review) is the author/funder, who has granted bioRxiv a license to display the preprint in perpetuity. It is made available under aCC-BY 4.0 International license.

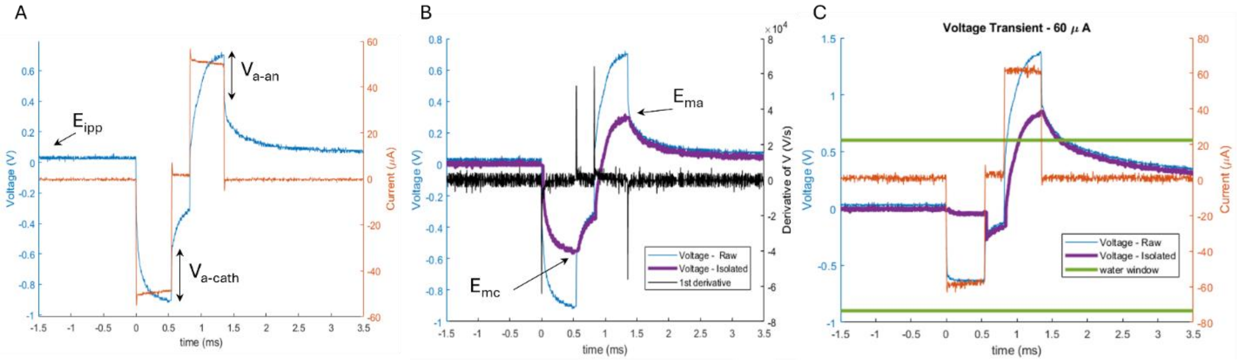


**Figure S3. Illustration of the process to obtain the isolated voltage transient waveform across the electrode-electrolyte interface from raw measurements to accurately calculate the charge injection capacity of the micro-electrodes from Smart Dura devices.** A) Raw data from biphasic current pulse

bioRxiv preprint doi: https://doi.org/10.1101/2025.02.26.640369; this version posted July 24, 2025. The copyright holder for this preprint (which was not certified by peer review) is the author/funder, who has granted bioRxiv a license to display the preprint in perpetuity. It is made available under aCC-BY 4.0 International license.

(red) and resulting voltage transient waveform recorded at the electrolyte from the Ag-AgCl reference electrode. B) First derivative (black) of raw voltage transient waveform (blue) used to identify the location of pulse edges ($t_{Va}$) from peaks to isolate the voltage exclusively across the electrode-electrolyte (cyan).
